# Effects of breaking up prolonged sitting following low and high glycaemic index breakfast consumption on glucose and insulin concentrations

**DOI:** 10.1007/s00421-017-3610-4

**Published:** 2017-05-12

**Authors:** Daniel P. Bailey, Benjamin D. Maylor, Charlie J. Orton, Julia K. Zakrzewski-Fruer

**Affiliations:** Institute for Sport and Physical Activity Research, School of Sport Science and Physical Activity, University of Bedfordshire, Polhill Avenue, Bedford, MK41 9EA United Kingdom

**Keywords:** Prolonged sitting, Sedentary behaviour, Physical activity, Postprandial glucose, Postprandial insulin, Cardiometabolic disease

## Abstract

**Purpose:**

Breaking up prolonged sitting can attenuate the postprandial rise in glucose and insulin. Whether such effects are dependent of the glycaemic index (GI) of the consumed carbohydrate is unknown. This study examined the acute effects of breaking up prolonged sitting following a low GI and a high GI breakfast on postprandial glucose and insulin concentrations.

**Procedures:**

Fourteen adult males aged 22.1 ± 1.2 years completed four, 4 h experimental conditions: high GI breakfast followed by uninterrupted sitting (HGI-SIT), low GI breakfast followed by uninterrupted sitting (LGI-SIT), high GI breakfast followed by 2 min activity breaks every 20 min (HGI-ACT), and low GI breakfast followed by 2 min activity breaks every 20 min (LGI-ACT). Positive incremental area under the curve (iAUC) for glucose and insulin (mean [95% CI]) for each 4 h experimental condition was calculated. Statistical analyses were completed using linear mixed models.

**Results:**

The sitting × breakfast GI interaction was not significant for glucose positive iAUC (*P* = 0.119). Glucose positive iAUC (mmol/L 4 h^−1^) was significantly lower in the activity breaks conditions than the uninterrupted sitting conditions (2.07 [2.24, 2.89] vs. 2.56 [1.74, 2.40], respectively, *P* = 0.004) and significantly lower in the low GI conditions than the high GI conditions (2.13 [1.80, 2.45] vs. 2.51 [2.18, 2.84], respectively, *P* = 0.022). Insulin concentrations did not differ between conditions (*P* ≥ 0.203).

**Conclusions:**

Breaking up prolonged sitting and lowering breakfast GI independently reduced postprandial glucose responses. This indicates that interrupting prolonged sitting and reducing dietary GI are beneficial approaches for reducing cardiometabolic disease risk.

## Introduction

Postprandial hyperglycaemia refers to an exaggerated elevation in blood glucose following the consumption of a meal. Elevated postprandial glucose is a significant risk factor for cardiometabolic disease (de Vegt et al. 2001); thus, interventions to reduce the rise in postprandial blood glucose may be important in disease prevention (Tuomilehto et al. 2001). In terms of dietary interventions, the glycaemic index (GI) quantifies the glycaemic response of carbohydrate-rich foods, with low GI foods resulting in a slow and steady release of glucose compared with HGI foods (Jenkins et al. 1981). Accordingly, acute experimental studies demonstrate a reduction in the postprandial glucose and insulin response to low GI compared with energy-matched high GI mixed meals (Galgani et al. 2006; Stevenson et al. 2009). Although low GI diets are commonly recommended to lower postprandial glycaemia (Buyken et al. 2010), adherence to this type of diet can be poor (Nisak et al. 2010). Furthermore, GI is not the single factor controlling postprandial glycaemia and there has been recent research interest in the potential impact of minimising prolonged periods of sitting during the postprandial period.

Independent of time spent in moderate-to-vigorous physical activity, spending a high amount of time sedentary is a significant risk factor for cardiometabolic disease (Wilmot et al. 2012). Acute experimental studies have observed reduced postprandial glucose and insulin concentrations when prolonged sitting is interrupted with 2 min bouts of light- or moderate-intensity activity every 20 min following the consumption of a standardised mixed liquid test drink (Bailey et al. 2016; Bailey and Locke 2015; Dunstan et al. 2012). However, an important limitation of these studies is that the liquid meals provided may not reflect habitual dietary patterns in free-living conditions. Furthermore, these studies have not considered or reported the GI of the meals provided. Indeed, it is possible that the attenuated rise in postprandial glucose and insulin concentrations from breaking up prolonged sitting depends on the degree of glycaemia induced by the meal. Specifically, there would be a greater ‘scope for improvement’ following meals inducing a greater glycaemic response. Thus, an understanding of whether breaking up prolonged sitting is effective in suppressing postprandial glucose responses to both low and high GI meals is required.

Evidence on the combined effects of breakfast GI and breaking up prolonged sitting on postprandial glycaemia would help inform combined activity and dietary approaches for preventing cardiometabolic disease. The primary aim of the present study was to examine the effect of breaking up prolonged sitting following the consumption of a high and a low GI breakfast on postprandial glucose and insulin responses in young adult males. It was hypothesised that breaking up prolonged sitting would reduce postprandial glucose concentrations, but that this reduction would be more pronounced following the consumption of a high GI breakfast compared with a low GI breakfast.

## Methods

### Study overview

This randomised four-way crossover design study was approved by the University of Bedfordshire School of Sport Science and Physical Activity Ethics Review Committee and conformed to the principles set out in the Declaration of Helsinki. All testing took place at the University of Bedfordshire Sport and Exercise Science Laboratories. After preliminary measures, participants completed four experimental conditions: (1) high GI breakfast + uninterrupted sitting, (2) low GI breakfast + uninterrupted sitting, (3) high GI breakfast + activity breaks, and (4) low GI breakfast + activity breaks. Each condition was separated by a 6–14 day washout period. The conditions were conducted in an incomplete counterbalanced order pre-determined using the Latin square method.

### Participants

Fourteen adult males aged 21–25 years gave informed consent to participate in the study following a written and verbal explanation of the nature and risks involved. Exclusion criteria were any blood borne disease, clinically diagnosed diabetes, taking glucose-lowering and/or lipid-lowering medication, known physical activity contraindications, major illness/injury (acute or chronic), or other health issues that may limit the ability to perform the necessary activity bouts.

### Sample size calculations

The primary outcome was postprandial glucose positive incremental area under the curve (iAUC). Our previous research reported a 16% reduction (effect size, *F* = 0.61) in 5 h postprandial glucose total area under the curve (AUC) when breaking up prolonged sitting with 2 min walking every 20 min versus uninterrupted sitting (Bailey and Locke 2015). Allowing for an intervention effect of 16% change in glucose positive iAUC, 10% within-group error variance, a within-person correlation of 0.6, 80% power, and an *α* of 0.05, it was estimated that 12 participants would be required for this complete four-treatment crossover design. Fourteen participants were recruited to allow for potential dropout.

### Preliminary measures

Stature was measured to the nearest 0.01 m using a stadiometer (Holtain Ltd., Crymych, Wales). Body mass was measured to the nearest 0.1 kg and body fat estimated to the nearest 0.1% using the Tanita BC-418 Segmental Body Composition Analyzer (Tanita Corp., Tokyo, Japan). Participants were then familiarised with use of the Borg Rating of Perceived Exertion (RPE) scale (Borg 1982) and completed a test to determine a moderate-intensity speed on a motorised treadmill (Woodway PPS55 Med-i, GmbH, Germany). In line with previous research (Bailey et al. 2016; Dunstan et al. 2012), the test began at a speed of 5 km h^−1^ and increased by 0.5 km h^−1^ every 2 min until an RPE of 12–14 (somewhat hard) was reached. Subsequently, the speed that elicited an RPE of 12–14 for each individual was used for the experimental conditions; the treadmill speeds ranged between 6.5 and 8.0 km h^−1^.

### Experimental protocol

**Fig. 1:**
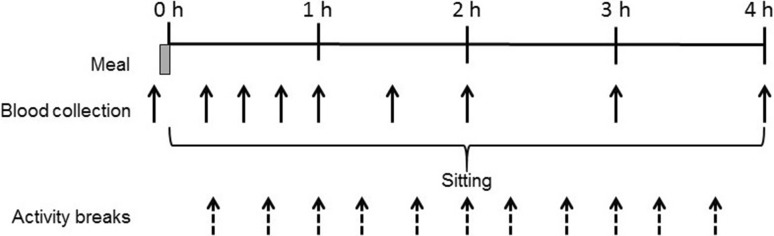
Schematic of study protocol

Figure 1 shows the experimental protocol. The 4 h experimental conditions were based on previous research that demonstrated suppressed postprandial glucose and insulin responses when breaking up prolonged sitting with 2 min activity every 20 min (Bailey and Locke 2015; Dunstan et al. 2012) and were as follows:

1. High GI breakfast + uninterrupted sitting (HGI-SIT): participants consumed a high GI breakfast followed by uninterrupted sitting at a desk, rising only to void.
2. Low GI breakfast + uninterrupted sitting (LGI-SIT): as HGI-SIT, but participants consumed a low GI breakfast.
3. High GI breakfast + activity breaks (HGI-ACT): participants consumed a high GI breakfast and then rose from the seated position every 20 min to complete 2 min bouts of moderate-intensity activity on a motorised treadmill, providing a total of 22 min activity (11 activity breaks). Participants returned to a seated position at a desk between activity breaks.
4. Low GI breakfast + activity breaks (LGI-ACT): as HGI-ACT, but participants consumed a low GI breakfast.

Participants were asked to refrain from exercise, alcohol, and caffeine in the 24 h before each experimental condition. Participants were asked to weigh and record all food and liquid intake in a food diary for 24 h before the first experimental condition and to replicate their diet (quantity and timings) prior to each subsequent experimental condition (Bailey et al. 2016). All participants attended the laboratory at ~0800 h after an overnight fast and were instructed to minimise activity during their commute. Upon arrival, participants rested for 30 min before a fasting blood sample was taken. A standardised high or low GI breakfast meal was then consumed within 15 min and the 4 h experimental condition commenced following the last mouthful of food. Participants were asked to void immediately prior to consumption of the breakfast meal and were then permitted to void when needed during the remainder of the conditions. The toilets were located ~30 m from the laboratory. Participants were supervised throughout the conditions to ensure adherence to the protocols and were permitted to watch DVDs, read books, magazines, or newspapers, talk, or work on a laptop computer. During sitting periods, participants were instructed to minimise excessive movement.

### Breakfast meal and water consumption

The high and low GI breakfast meals provided 1 g carbohydrate kg body mass^−1^ for each participant. The meals were matched for macronutrient content (70% carbohydrate, 16% fat and 14% protein), energy and fluid, but the high GI breakfast contained 40% less fibre than the low GI breakfast. The high GI breakfast consisted of cornflakes, skimmed milk, white bread and strawberry jam. The low GI breakfast consisted of muesli, semi-skimmed milk, apple and peaches. The GI values for each food item were obtained from the International Tables of Glycaemic Index and Glycaemic Load Values 2008 (Atkinson et al. 2008) and breakfast GI was calculated using weighted means of the GI values for the component foods (Wolever and Jenkins 1986). The calculated GI values of the high and low GI breakfasts were 78 and 47, respectively. During the first experimental conditions, consumption time was recorded and participants were asked to replicate this consumption time as closely as possible in each subsequent condition. Water was provided ad libitum during the first condition and the volume consumed was replicated during each subsequent condition.

### Blood collection and biochemistry

During each experimental condition, capillary blood samples were collected by finger prick (Haemolance Plus Lancet, Prospect Diagnostics, Dronfield, UK). Capillary rather than venous blood sampling is preferred for reliable GI testing (Wolever et al. 2003). The first sample was taken in a fasted state at −15 min and followed by subsequent samples at 15, 30, 45, 60, 90, 120, 180 and 240 min during the 4 h postprandial period. Hourly blood samples were obtained immediately prior to the activity breaks (i.e., for 60, 120, 180 and 240 min). At each time point, ~600 μL of whole blood was collected into two EDTA-containing microvettes (Microvette CB300 EDTA, Sarstedt Ltd, Leicester, UK). Blood glucose was analysed immediately using the YSI 2300 STAT plus glucose and lactate analyzer (YSI Inc., Yellow Springs, OH, USA) using 30 μL of whole blood from one microvette. The YSI uses a steady state measurement methodology, where membrane based glucose oxidase catalyses the oxidation of glucose to gluconic acid and hydrogen peroxide. The difference between the sample generated plateau current and the initial baseline current is proportional to the glucose concentration. The YSI was calibrated at the start of every experimental condition and every 45 min thereafter. The remaining whole blood (~570 μL) was centrifuged at 2500×*g* for 5 min (Heraeus Pico 17, Thermo Scientific, Loughborough, UK). Subsequently, the plasma was extracted and stored at −80 °C for later batch analysis of insulin. Plasma insulin concentration was measured in duplicate using an enzyme-linked immunosorbent assay (Mercodia, Uppsala, Sweden). To eliminate inter-assay variation, samples from each participant were analysed in the same run. The intra-assay coefficient of variation was 9.2%.

### Calculation of outcome variables

Positive iAUC and total AUC were calculated for glucose and insulin for each 4 h condition using the trapezoidal method. For the positive iAUC calculation, any value below baseline (fasting) was treated as a baseline value; it has been suggested that this method more accurately describes glycaemic responses to food compared with total AUC (Le Floch et al. 1990). Use of this method also permits direct comparisons with previous research (Bailey et al. 2016).

### Statistical analyses

Statistical analyses were completed using SPSS version 22.0 (SPSS Inc., Armonk, N.Y., USA). Quantile–Quantile plots were used to check the normality assumption of the results obtained for each of the conditions. This method was preferred over null hypothesis significance testing to check statistical assumptions (Grafen and Hails 2002). Linear mixed models were used to evaluate the main effect of sitting (uninterrupted sitting vs. activity breaks) and breakfast GI (high GI vs. low GI) and the sitting × breakfast GI interaction for the dependent variables. Condition and covariates (age, fasting glucose/insulin concentrations, and body fat%) were fixed factors and subjects were random factors within the models. When there was a significant sitting × breakfast GI interaction, post hoc comparisons examined differences between the four individual conditions using Sidak correction for multiple comparisons. Cohens’ d effect sizes were calculated if *P* ≤ 0.200 to examine whether the magnitude of differences between conditions was potentially meaningful; 0.2, 0.5 and 0.8 indicated a small, medium or large effect, respectively (Cohen 1988). Data are presented as mean (95% confidence interval [CI]) unless stated otherwise. The two-tailed alpha level for significance testing was set as *P* ≤ 0.05.

## Results

Descriptive characteristics of the participants are reported in Table 1. Eight participants had a body mass index (BMI) ≥ 25 kg/m^2^ and six had a BMI < 25 kg/m^2^. Thirteen participants had a low body fat level (i.e. <25%) (Shah and Braverman 2012). Although one participant had a high body fat level (≥25%), their fasting glucose concentration was normal (<6.1 mmol/L) (World Health Organization 2006) and their postprandial glucose response was lower than ten of the other participants. Therefore, this participant was not excluded from the analyses.

**Table 1:** Descriptive participant characteristics (*n* = 14)

	Mean ± SD
Age (years)	22.1 ± 1.2
Height (cm)	176 ± 6.6
Body mass index (kg/m^2^)	25.0 ± 3.1
Body fat%	17.2 ± 5.5

*SD* standard deviation

Table 2 shows fasting and AUC values for glucose and insulin concentrations for each condition. Fasting glucose concentrations did not differ significantly between conditions and the sitting × breakfast GI interaction was not significant. All participants had a fasting glucose concentration of ≤4.77 mmol/L, which is within the normal range (World Health Organization 2006). Although the sitting × breakfast GI interaction for postprandial glucose iAUC did not reach significance, there was a large effect size for the difference between HGI-SIT and HGI-ACT (*d* = 0.97), HGI-SIT and LGI-ACT (*d* = 1.14), and HGI-SIT and LGI-SIT (*d* = 0.83). There was a small effect size for the difference between LGI-SIT and LGI-ACT (*d* = 0.31), LGI-SIT and HGI-ACT (*d* = 0.14), and LGI-ACT and HGI-ACT (*d* = 0.17). There was a significant main effect of sitting for glucose positive iAUC, with concentrations in the activity breaks conditions being 21% lower than the uninterrupted sitting conditions (2.07 mmol/L 4 h [1.74, 2.40] vs. 2.56 mmol/L 4 h [2.24, 2.89], respectively). The effect size for this difference was large (*d* = 0.76). The main effect of breakfast GI was also significant, with glucose positive iAUC concentrations 16% lower in the low GI breakfast conditions than the high GI breakfast conditions (2.13 mmol/L 4 h [1.80, 2.45] vs. 2.51 mmol/L 4 h [2.18, 2.84], respectively). There was a medium effect size for this difference (*d* = 0.59).

**Table 2:** Biochemical values for each condition

	High GI breakfast + uninterrupted sitting	Low GI breakfast + uninterrupted sitting	High GI breakfast + activity breaks	Low GI breakfast + activity breaks	*P* value for main effect of sitting	*P* value for main effect of breakfast GI	P value for sitting × breakfast GI interaction
Fasting blood glucose (mmol/L)	4.38 (4.22, 4.53)	4.37 (4.22, 4.52)	4.39 (4.23, 4.54)	4.31 (4.16, 4.46)	0.735	0.535	0.625
Fasting plasma insulin (μU/mL)	7.38 (5.80, 8.97)	8.76 (7.17, 10.35)	8.55 (6.96, 10.14)	6.76 (5.17, 8.35)	0.531	0.750	0.020
Positive iAUC for blood glucose (mmol/L 4 h)	2.88 (2.49, 3.28)	2.25 (1.85, 2.64)	2.13 (1.74, 2.53)	2.01 (1.61, 2.40)	0.004	0.022	0.119
Positive iAUC for plasma insulin (μU/mL 4 h)	60.31 (48.61, 72.01)	55.05 (43.26, 66.84)	58.09 (46.36, 69.83)	52.03 (40.18, 63.89)	0.554	0.203	0.932
Total AUC for blood glucose (mmol/L 4 h)	20.25 (19.83, 20.68)	19.28 (18.85, 19.70)	19.51 (19.08, 19.94)	18.80 (18.38, 19.22)	0.001	<0.001	0.440
Total AUC for plasma insulin (μU/mL 4 h)	88.56 (76.62, 100.51)	83.45 (71.41, 95.48)	88.02 (76.04, 100.00)	79.78 (67.67, 91.87)	0.639	0.141	0.741

Data presented as mean (95% CI)

*GI* glycaemic index, *iAUC* incremental area under the curve, *AUC* area under the curve

**Fig. 2:**
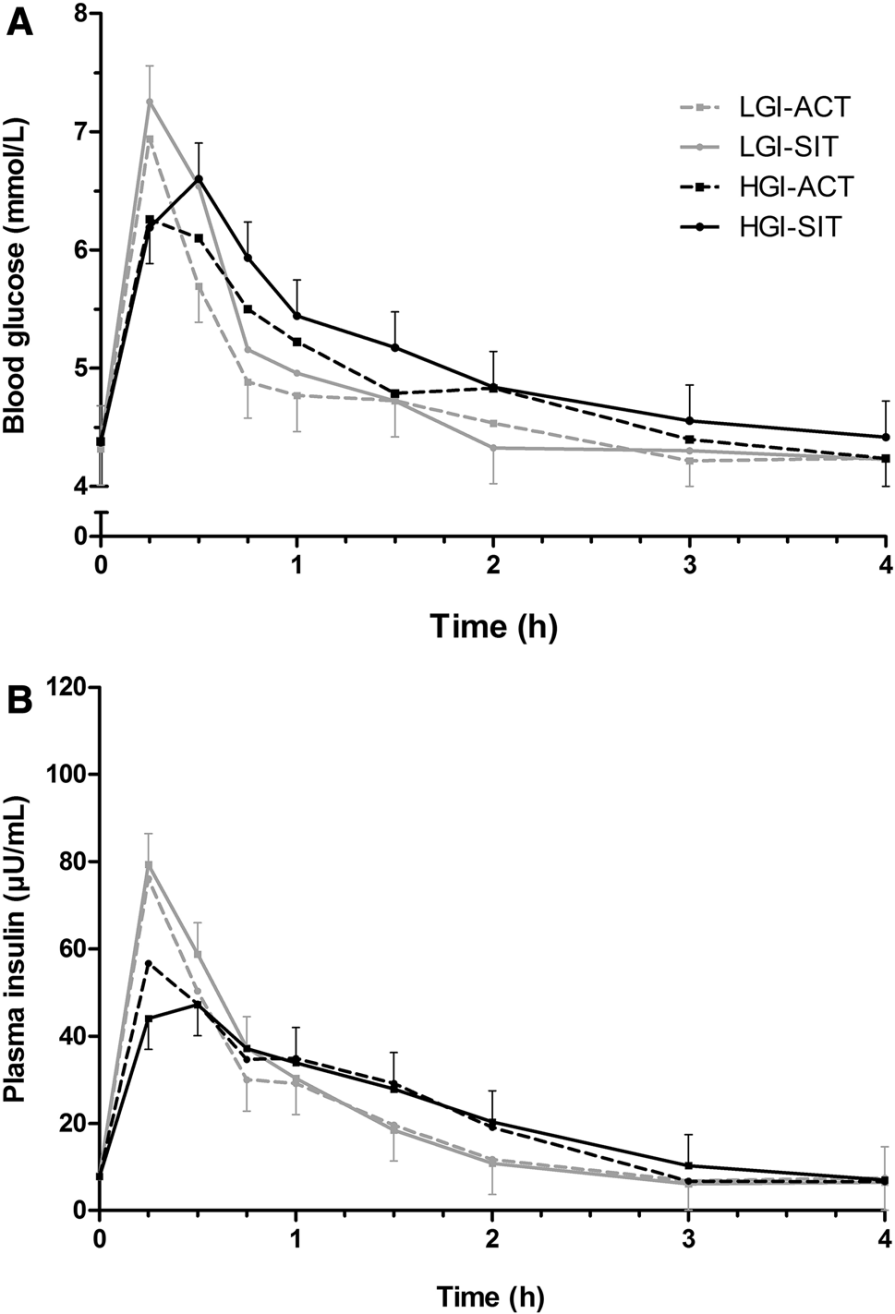
Changes in blood glucose (**a**) and plasma insulin (**b**) concentrations during the low glycaemic index (GI) breakfast + activity breaks (LGI-ACT), low GI breakfast + uninterrupted sitting (LGI-SIT), high GI breakfast + activity breaks (HGI-ACT), and high GI breakfast + uninterrupted sitting (HGI-SIT) conditions. Data are mean and 95% confidence interval. Some *error bars* have been omitted for clarity

Analysis of glucose total AUC also revealed that the sitting × breakfast GI interaction was not significant (Table 2). There was a main effect for sitting and for breakfast GI, with glucose total AUC being significantly lower in the activity breaks conditions than the uninterrupted sitting conditions (19.15 mmol/L 4 h [18.79, 19.52] vs. 19.77 mmol/L 4 h [19.40, 20.13], respectively; *d* = 0.86) and significantly lower in the low GI breakfast conditions than the high GI breakfast conditions (19.04 mmol/L 4 h [18.67, 19.40] vs. 19.88 mmol/L 4 h [19.52, 20.24], respectively; *d* = 1.17). Glucose concentrations over time for each trial are shown in Fig. 2.

The sitting × breakfast GI interaction for fasting insulin concentration was significant (Table 2). Fasting insulin concentration was higher in LGI-SIT than LGI-ACT (*P* = 0.037), but there were no significant differences between any of the other conditions (*P* ≥ 0.059). The sitting × breakfast GI interaction and the main effects of sitting and breakfast GI for postprandial insulin positive iAUC and total AUC were all non-significant (Table 2). Insulin concentrations over time for each trial are shown in Fig. 2.

## Conclusions

This is the first study to assess the acute postprandial glucose and insulin response to breaking up prolonged sitting following meals differing in GI. The present study found that breaking up prolonged sitting with moderate-intensity activity acutely lowers the postprandial glucose response following a low or high GI breakfast. Similarly, lowering the GI of breakfast acutely lowered the postprandial glucose response regardless of whether postprandial sitting was interrupted with activity breaks or not. There was minimal evidence that combining these two interventions had a cumulative added impact on lowering postprandial glycaemia.

The finding that postprandial glucose responses are lowered when sitting is interrupted with moderate-intensity activity is synonymous with previous experimental research (Bailey et al. 2016; Dunstan et al. 2012; Peddie et al. 2013). These studies have observed benefits in postprandial glucose despite differences in the age, weight status, and metabolic status of the participants, and methodological differences in the duration of the experimental conditions, duration and frequency of interruptions to sitting time, and the test meal frequency, timing, and calorie content. The present study contributes to current knowledge by demonstrating that the benefits of breaking up prolonged sitting are observable following consumption of meals differing in GI.

In contrast to previous research in normal weight, overweight and obese adults (Dunstan et al. 2012; Peddie et al. 2013), the present study did not observe reductions in postprandial insulin responses to breaking up sitting time. Dunstan et al. (2012) studied older (45–65 years) males and females and it is possible that the younger male participants in the present study were able to increase their glucose disposal in response to the activity breaks via insulin-independent pathways, such as increased translocation of the intracellular glucose transporter protein GLUT-4 (Latouche et al. 2013), increased permeability of muscles cells to glucose (Wallberg-Henriksson et al. 1988), and increased carbohydrate oxidation (Peddie et al. 2013) that may occur in response to muscular contractions. However, Peddie et al. (2013) studied males and females of a similar age to the present study. It is possible that the longer observation period, more frequent test meal consumption and differences in test meal composition could explain the lowered insulin response with interruptions to sitting time in the study by Peddie et al. (2013), which was not observed in the present study.

In line with previous research using similar mixed breakfast meals (Stevenson et al. 2009; Zakrzewski et al. 2012), the high GI breakfast induced a larger glucose response compared with the low GI breakfast. Somewhat unexpectedly, the postprandial peak in glucose and insulin concentrations was higher in the low GI compared with the high GI breakfast conditions. Thus, the reduced glucose iAUC with low GI breakfast consumption was due to lower glucose concentrations that occurred after the postprandial peak (i.e., from 15 to 240 min). Although there is evidence that GI predicts the peak in blood glucose for individual food items (Brand-Miller et al. 2009), GI values are defined by glucose iAUC rather than the peak blood glucose concentration after food consumption. Our data suggests that predicted GI values for mixed meals may not necessarily reflect expected differences in peak glucose concentrations. Thus, further research is required to confirm the applicability of GI values from single food items to mixed meals when considering peak glucose concentrations. Despite the higher glucose response to the high GI breakfast, the postprandial insulin iAUC was not different between the breakfast meals. Thus, reduced insulin-stimulated disposal of glucose may have contributed to the higher overall glycaemic response to the high GI breakfast. Although the breakfasts in the present study were matched for carbohydrate, fat and protein, it should be noted that the higher fibre content in the low GI breakfast may have contributed to the lower glycaemic response by acting as a physical barrier and delaying access of digestive enzymes to breakdown carbohydrate (Jenkins et al. 1981; Nuttall 1993). With this in mind, it may be more appropriate to recommend low GI high-fibre breakfasts (rather than LGI breakfasts per se) for reducing postprandial glycaemia, but not necessarily for reducing peak glucose concentrations.

The non-significant sitting by breakfast GI interaction indicates that these interventions have independent effects on lowering postprandial glycaemia. Nevertheless, it should be highlighted that breaking up sitting lowered the glucose response by 30% following high GI breakfast consumption, but only by 11% following low GI breakfast consumption. The larger effect size for breaking up sitting following the high GI breakfast compared with the low GI breakfast could potentially be clinically meaningful, suggesting that breaking up sitting could have more pronounced benefits following higher GI breakfasts. With this in mind, the sitting by breakfast GI interaction may not have been significant due to a lack of statistical power in the present study. The relatively high glucose response to the high GI breakfast could explain why breaking up sitting resulted in a more pronounced reduction in postprandial glucose compared with a low GI breakfast. This could suggest that dietary GI may be important in determining the magnitude of response to interventions involving breaking up prolonged sitting. Thus, future research on the metabolic effects of breaking up prolonged sitting should consider and report the GI content of the meals provided to participants.

It is unknown whether the magnitude of the observed reductions in postprandial glucose in response to breaking up prolonged sitting in this study equates to clinical significance in a young adult population. Nevertheless, postprandial excursions in glucose are a cardiometabolic disease risk factor in healthy, nondiabetic individuals (Levitan et al. 2004), suggesting that the findings of this study have meaningful implications. Furthermore, reductions in dietary GI remain an important public health target for the prevention of cardiometabolic disease (Buyken et al. 2010). However, it should be emphasised that the findings reported here are based on young, healthy males and may not apply directly to treat clinical populations.

This study has several potential limitations. The sample included young adult males and the generalisability of the findings to other population groups is uncertain. This study compared the effects of consuming different GI meals and breaking up prolonged sitting over a period of 4 h following a single meal. Responses to multiple meals consumed across the course of the day thus cannot be inferred, nor can the long term responses to the consumption of a low GI diet or breaking up prolonged sitting. Participants were asked to refrain from exercise 24 h prior to experimental conditions. However, an acute bout of exercise may enhance insulin sensitivity for up to 48 h (Mikines et al. 1988); therefore, future studies should consider asking participants to refrain from exercise for a minimum of 48 h prior to experimental conditions. Fasting insulin concentrations were higher in the low GI + uninterrupted sitting condition than the low GI + activity breaks condition. However, this difference was small and the positive iAUC calculations account for differences in baseline (fasting) values between conditions (Le Floch et al. 1990). In addition, all analyses were adjusted for fasting glucose and insulin concentrations. Another potential limitation of the study is that the GI values of the breakfast meals used were predicted, and there is evidence that the predicted GI of mixed meals does not always reflect the GI of individual food items (Flint et al. 2005). Nevertheless, the low GI breakfast induced significantly lower glucose positive iAUC compared with the high GI breakfast, as predicted. Finally, it is unknown whether the peak in postprandial glucose observed at 15 min in the low GI conditions could be attenuated by breaking up prolonged sitting as the first interruption in sitting time in the present study did not occur until 20 min.

In conclusion, breaking up prolonged sitting with moderate-intensity activity acutely lowers the postprandial glucose response following consumption of a low and high GI breakfast in young adult males. Similarly, lowering the GI of breakfast reduced the postprandial glucose response independent of whether the postprandial period was interrupted with activity breaks or not. These findings emphasise the importance of avoiding prolonged periods of sitting and high GI breakfasts to reduce cardiometabolic disease risk.

## Abbreviations

AUC: Area under the curve
BMI: Body mass index
CI: Confidence interval
GI: Glycaemic index
HGI-ACT: High glycaemic index breakfast + activity breaks
HGI-SIT: High glycaemic index breakfast + uninterrupted sitting
iAUC: Incremental area under the curve
LGI-ACT: Low glycaemic index breakfast + activity breaks
LGI-SIT: Low glycaemic index breakfast + uninterrupted sitting
RPE: Rating of perceived exertion
